# Early diagnosis of rats with acute myocardial infarction by measurement of brain natriuretic peptide

**DOI:** 10.3892/etm.2013.964

**Published:** 2013-02-18

**Authors:** JIAN LI, FANG-FANG YIN, YING-LONG HOU

**Affiliations:** Department of Cardiology, Qianfo Mountain Hospital Affiliated with Shandong University, Jinan, Shangdong 250014, P.R. China

**Keywords:** brain natriuretic peptide, acute myocardial infarction, early diagnosis, model animal, Wistar rat

## Abstract

The aim of this study was to detect early changes (within 1–4 h) in the brain natriuretic peptide (BNP) levels of rats with acute myocardial infarction (AMI). A total of 35 Wistar rats were established as models of AMI and 30 sham-operated rats were used as the control group. The myocardia of the two groups were observed using a transmission electron microscope (TEM) prior to and following surgery. A double-antibody sandwich enzyme-linked immunosorbent assay (ELISA) was used to detect the serum BNP and cardiac troponin I (cTnI) concentrations before and 1–4 h after surgery. Following the successful establishment of the AMI models, serum BNP concentrations were significantly increased within 1–4 h compared with the values prior to surgery and with those of the control group (all P<0.01). The serum BNP concentration reached its highest level 2 h after AMI (532.25±15.16 ng/l). No significant changes were observed in the cTnI serum levels of the AMI group within 1–4 h compared with the values before AMI and those in the control group (all P>0.05). In the 1–4 h following the establishment of the AMI model, significant positive correlations were identified between the serum BNP concentrations and the size of the AMI and the most marked correlation occurred 2 h after AMI (r=0.72, P<0.05). No significant differences were noted in the serum concentrations of BNP and cTnI in the control group prior to and following the sham surgery (all P>0.05). BNP may be used as a blood marker for the early diagnosis of AMI, particularly 1–4 h after the onset of AMI, and to predict the size of the infarct area.

## Introduction

Brain natriuretic peptide (BNP) has been a focus of cardiac research since it was discovered by Sudoh *et al* in 1988 (1). The BNP test is used clinically in the early diagnosis of heart failure (HF), risk stratification in HF, diagnosis of acute dyspnea, prognosis and assessment of HF and screening of high-risk groups, as well as risk stratification in acute coronary syndrome (ACS) and the assessment of the treatment of HF (2–13). However, at present, opinions concerning the value of BNP in the early diagnosis of acute myocardial infarction (AMI) differ (14–19). The variations in BNP levels and the mechanism of action of BNP at the early stage of AMI remain unclear, particularly during the first 4 h following onset. To clearly understand the trend in the changes of BNP level during the early stage of AMI, particularly within the first 4 h following onset, animal models were established in the present study. The changes in BNP levels in the first 4 h of AMI were determined and compared with the levels of cardiac troponin I (cTnI).

## Materials and methods

### Experimental animals

A total of 66 Wistar rats, both male and female, provided by the Animal Experiment Center of Shangdong University (Jinan, China), were used in the study. The study was performed in strict accordance with the recommendations in the Guide for the Care and Use of Laboratory Animals of the National Institutes of Health. The animal use was reviewed and approved by the Institutional Animal Care and Use Committee (IACUC) of the Qianfo Mountain Hospital Affiliated with Shandong University. Body weights were between 316 and 381 g and the average weight was 337.14±20.55 g. A model of AMI was established in 36 rats (the AMI group), while 30 sham-operated rats were used as the control group. Ultrastructural observations in the myocardia were performed after surgery in the two groups. One rat died during surgery in the AMI group.

### Animal models

Thirty-six experimental rats were intraperitoneally anesthetized with 3.6% chloral hydrate (10 ml/kg) and then bound on a rat table. The trachea was separated and intubated to connect to a small animal ventilator supporting a breath rate of 50–60 breaths per minute and a tidal volume of 4–5 ml per breath. Needle electrodes connected to an electrocardiography device were inserted subcutaneously into the limbs and a standard electrocardiogram was recorded. A left sternal border thoracotomy through the bed of the second to fourth rib was performed. The pericardium was opened, exposing the heart and blood vessels on the left ventricular surface. Using the left main coronary artery as a marker, needles were inserted 2 mm under the root of the left atrial appendage and a 3/0 silk thread was used to pass through the myocardial surface. The needle was withdrawn next to the pulmonary cone. The thread ring was knotted at both ends of the ligature, tightening the ligature to cause myocardial infarction. ST-segment elevation in electrocardiogram represented the successful establishment of AMI models. Penicillin and streptomycin were administered postoperatively to prevent infection. One rat died during surgery. A total of 35 rats successfully underwent the experimental surgery and 30 rats underwent a sham surgery, which involved the same procedure as that described above, but without the coronary artery ligation.

### Biochemical indicators

Jugular venous blood (2 ml) was extracted from experimental (n=35) and control groups (n=30) before and 1, 2, 3 and 4 h after the surgery. A desktop room-temperature centrifuge (UNIVERSAL Type 320R; Weifang Yuhua Medical Equipment Co., Ltd, WeiFang, China) was used to centrifuge the blood at 4,500 rpm for 10 min. The serum was separated and stored at −20°C. A double-antibody sandwich enzyme-linked immunosorbent assay (ELISA) was used to detect the concentrations of BNP and cTnI. The kit was purchased from Dalian Pan-State Chemical Technology Development Co., Ltd, Dalian, China.

### AMI size

Rats were anesthetized and sacrificed following the surgery and analysis. Hearts were removed and had an average weight of 1.04±0.04 g. The main left coronary artery was ligated. The aorta was retrograde perfused with 2–3 ml solution containing 0.5% Evans blue dye. The non-blue-stained ischemic area and the blue-stained non-ischemic area were separated. After being drawn through filter paper, the non-blue-stained ischemic myocardium was frozen at −20°C for 20 min and then underwent horizontal long axis slicing at a thickness of 1–2 mm. The slices were placed in 1% triphenyl tetrazolium chloride (TTC) in phosphate buffer solution (pH 7.4) and incubated at 37°C for 20 min. At this point, the necrotic zone (NZ) was gray and the non-necrotic zone (NNZ) scarlet. The NZ and NNZ were separated, drawn through filter paper and weighed on an AB104-N electronic balance. The infarct size was defined as the ratio of the weight of the NZ to that of the ischemic zone (the sum of NZ and NNZ).

### Myocardial ultrastructure

Following the surgery, the rat myocardia were fixed with 3% glutaraldehyde, then fixed with 1% osmium tetroxide and dehydrated conventionally in an ethanol gradient. Through epoxy resin embedding and deployment of a hardener, accelerator and growth agent, ultra-thin sections with a thickness of 70 nm were cut by ultramicrotomy and stained with uranyl acetate and lead citrate solution. Changes in the myocardial ultrastructure were observed with a JEM-1200EX transmission electron microscope (TEM). The required area was selected for image capture. The myocardia of 8 normal rats were observed with the TEM as the normal control prior to surgery.

### Statistical analysis

SPSS 11.0 software was used to analyze the data. Data were expressed as the mean ± standard deviation. The paired t-test was used for comparisons of before and after surgery and linear correlation analysis was utilized to analyze the correlation among the variables. P<0.05 was considered to indicate a statistically significant difference.

## Results

### Serum BNP concentrations

The serum BNP concentrations were significantly higher 1 h after the successful establishment of the AMI model than prior to surgery (P<0.01). The BNP level reached a peak after 2 h and remained significantly higher in the first 4 h (all P<0.01). The post-surgery serum BNP concentrations of the rats at 1–4 h were significantly higher than those in the control group (all P<0.01; Table I).

### cTnI serum level

No statistically significant differences were observed between the serum cTnI concentrations in the AMI group before and 1–4 h after surgery (P>0.05). The serum cTnI concentrations of the AMI group before and 1–4 h after surgery were not significantly different from those of the control group (P>0.05). In addition, the differences between the serum cTnI concentrations of the control group before and 1–4 h after surgery were not statistically significant (P>0.05; Table II).

### Correlation analysis

The serum BNP concentrations showed a significant positive correlation with the infarct size in the AMI group at 1, 2, 3 and 4 h after surgery (r=0.34, P<0.05; r=0.72, P<0.05; r=0.57, P<0.05; and r=0.48, P<0.05, respectively). The serum cTnI concentrations of the AMI group at 1, 2, 4 and 4 h after surgery were not significantly correlated with the myocardial infarct range (r=–0.07, P>0.05; r=–0.05, P>0.05; r=–0.09, P>0.05; and r=–0.05, P>0.05, respectively).

### Myocardial ultrastructure

**Figure 1:**
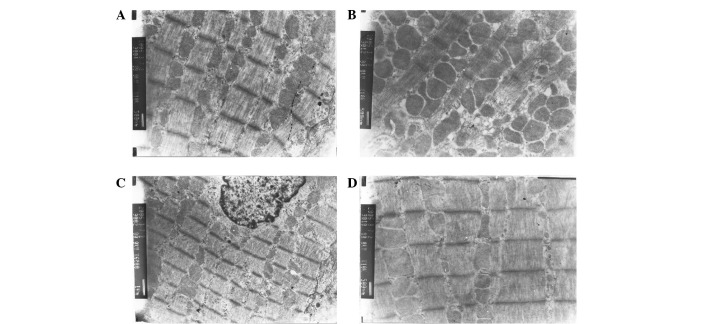
Myocardial ultrastructure. (A) Normal myocardial fibers of rats prior to surgery (TEM, x10,000). (B) Variations in the size and shape, hyperplasia and muscle fracture of the mitochondria of rats following AMI (TEM, x10,000). (C) Normal myocardial fiber prior to surgery in the control group (TEM, x6,000). (D) Normal myocardial fiber following surgery in the control group with clear regular structure and rows of mitochondria among the sarcomeres (TEM, x10,000). TEM, transmission electron microscopy; AMI, acute myocardial infarction.

The cardiac muscle fibers of the rats showed a clear regular structure when the myocardium was viewed under a microscope prior to surgery (Fig. 1A). At 4 h after the establishment of the AMI model, the myocardial mitochondria of the rats varied in size and shape and exhibited hyperplasia and muscle fracture (Fig. 1B). The cardiac muscle fibers of the rats in the control group prior to and following surgery were normal, with a clear regular structure and rows of mitochondria among the sarcomeres (Fig. 1C and D).

## Discussion

The rapid and accurate diagnosis in the early stage of AMI, particularly within 4 h of the onset, is significant for timely processing by clinicians and the prognosis of patients. The increase in the levels of the cardiac marker cTnI is only significant 4 h after the onset of AMI. Therefore, within 4 h of the onset of AMI, the diagnostic value of cTnI is limited (20,21). Studies have identified that BNP levels increase significantly at the early stage of non-ST elevation myocardial infarction (NSTEMI), which may be useful for the early diagnosis of AMI. BNP may be a complementary indicator for the early diagnosis of AMI (14). However, there has been no systematic review of animal studies of AMI to demonstrate this.

BNP is a member of the natriuretic peptide family, identified following the first description of atrial natriuretic peptide. BNP was named as such following its discovery in porcine brains by Sudoh *et al* (1) in 1988. BNP is a cardiac hormone predominantly synthesized in and secreted from the ventricles and has the effects of promoting natriuresis, diuresis and a marked dilation of blood vessels, as well as acting against the vasoconstriction of the renin-angiotensin-aldosterone system. The natriuretic peptide system is activated when cardiac dysfunction occurs. Ventricular overload contributes to its release. The BNP concentration is useful for to determining heart function and assessing the prognosis of patients with HF.

Following the study by Bassan *et al* (14), clinicians gained a new understanding of BNP. Since the previous knowledge of BNP had been limited to the risk stratification of HF prognoses, the diagnostic value of BNP in acute myocardial ischemia at the early stage had not been investigated. The study by Bassan *et al* demonstrated that BNP may be used as an early indicator of NSTEMI. Among chest pain patients without ST-segment elevation, the detection of BNP may help to filter out cases of NSTEMI. BNP levels significantly increased 2 h after NSTEMI while those of creatine kinase-MB (CK-MB) and cTnI did not. This study also revealed that the increase in BNP levels occurred earlier than the increases in CK-MB and cTnI, which aided the identification of patients in the early stage (chest pain within 4 h) of AMI without ST-segment elevation. For patients with chest pain without ST-segment elevation who were not in the diagnostic window for the indicators of myocardial necrosis, CK-MB and cTnI (4 h), the significant increase in BNP levels suggested the possibility of the onset of AMI. These patients required closer and continued monitoring of the changes in CK-MB or cTnI.

The study by Bassan *et al* was performed on NSTEMI patients. There has been no systematic animal study to determine whether the same or similar conclusions may be drawn under strict experimental conditions. The results of the present study showed that serum BNP concentrations were significantly increased 1 h after the successful establishment of the AMI model (compared with those in the control group prior to and following surgery, all P<0.01). The BNP levels reached a peak after 2 h, which was 9-fold higher than the normal value prior to surgery and persisted at a high level for 4 h after surgery. This was due to the accelerated secretion of BNP, which was mainly concentrated in the fringes of the border areas between the infarction and non-infarction regions. The mechanical tension of the ventricle wall in this area was the strongest.

Changes in the tension of the ventricular wall local to the infarction may be reflected by the BNP level. Since the tension is caused by the size of the infarction, morphological changes in the left ventricle, myocardial mechanical stress and other factors, the acceleration of ventricular wall tension may rapidly stimulate BNP gene expression, causing a large amount of synthesized BNP to be secreted into the blood. The study by Ogawa *et al* (22) showed that the ventricle was the key location for synthesizing and secreting BNP. The change in ventricular wall tension may stimulate the ventricle to synthesize and secrete BNP. Regional left ventricular diastolic and/or systolic dysfunction is a sign of myocardial acute or persistent ischemia. When acute coronary artery occlusion and sublethal myocardial ischemia occur, the ventricle may release BNP. Therefore, plasma BNP level measurements may predict the onset of AMI. The results of the present study demonstrated that BNP levels at the early stage of AMI (within 4 h) showed significant increases prior to notable changes in cTnI being exhibited. The BNP levels reached a peak at 2 h and then decreased. From 1 to 4 h, the serum BNP concentrations showed a significant positive correlation with the infarct size of the AMI. This was due to the ventricular muscle cells producing and secreting more BNP. The secretion of BNP was basically regulated by the increase in ventricular wall tension and left ventricular extension. The increase in ventricular wall tension rapidly stimulated BNP gene expression and a large amount of synthesized BNP was secreted into the blood. The ventricular wall tension in areas bordering the infarction and non-infarction regions was the strongest. The secretion of BNP was concentrated in this area and may exactly reflect the change of ventricular wall tension in local infarctions. The ventricular wall tension is affected by the size of the infarction, morphological changes in the left ventricle and other factors, so the determination of serum BNP concentrations in rats after AMI may predict the size of AMI. The present study showed that the BNP serum level in rats 2 h after surgery had the most marked correlation with AMI size (r=0.72, P<0.05). This was due to the myocardium showing significant necrosis 2 h after the establishment of the AMI model. The first indication was diastolic dysfunction, which stimulated ventricular synthesis and BNP release. There was a significant correlation between the BNP level and AMI size at every time point between 1 and 4 h after surgery. Therefore, measuring plasma BNP levels may predict the infarct size of AMI. According to the results observed under a TEM, the establishment of the AMI model in rats was successful. Compared with the data from before the surgery, the myocardial fibers of rats were broken 4 h after surgery, euchromatin in the nucleus was significantly reduced, mitochondria varied in size and shape, hyperplasia occurred and an obvious notch was present in the nucleus. These observations were due to the effects of the AMI.

The results of the present study demonstrate that BNP levels, which increase earlier than cTnI levels in the early stage of AMI, may be used as a blood marker for the early diagnosis of AMI and to predict infarct size. cTnI is a specific indicator of AMI, but is not particularly sensitive. No significant increase in the serum cTnI level was observed 4 h after the induction of AMI in rats; similar observations have been made previously for CK-MB. This clearly limits the diagnostic value of cTnI and CK-MB in the early phase, 4 h after AMI. The detection of BNP levels at this time is of value. Excluding other factors, BNP may be used as an early indicator for the rapid diagnosis of AMI.

## Figures and Tables

**Table I:** Changes in the serum BNP concentrations of rats before and after surgery (ϱ/ng/l, mean ± standard deviation).

Group	n	Pre-surgery	Post-surgery			
			1 h	2 h	3h	4 h
AMI	25	53.14±7.49	312.33±14.51^ab^	520.66±16.85^ab^	486.51±15.73^ab^	422.11±15.23^ab^
Control	20	53.12±7.46	55.33±8.51	54.14±7.85	52.18±8.34	55.37±8.72

a P<0.01 vs. pre-surgery,

b P<0.01 vs. control group. BNP, brain natriuretic peptide; AMI, acute myocardial infarction.

**Table II:** Changes in the cTnI serum levels of rats before and after surgery (*ϱ*/μg/l, mean ± standard deviation).

Group	n	Pre-surgery	Post-surgery			
			1 h	2 h	3 h	4 h
AMI	25	0.43±0.06	0.51±0.08	0.45±0.08	0.52±0.09	0.61±0.08
Control	20	0.43±0.05	0.47±0.09	0.46±0.09	0.51±0.07	0.58±0.07

cTnI, cardiac troponin I; AMI, acute myocardial infarction.
